# Application of Machine Learning Algorithms to Predict Lymph Node Metastasis in Early Gastric Cancer

**DOI:** 10.3389/fmed.2021.759013

**Published:** 2022-01-18

**Authors:** HuaKai Tian, ZhiKun Ning, Zhen Zong, Jiang Liu, CeGui Hu, HouQun Ying, Hui Li

**Affiliations:** ^1^Department of General Surgery, First Affiliated Hospital of Nanchang University, Nanchang, China; ^2^Department of Gastrointestinal Surgery, Second Affiliated Hospital of Nanchang University, Nanchang, China; ^3^Department of Day Ward, First Affiliated Hospital of Nanchang University, Nanchang, China; ^4^Department of Nuclear Medicine, Jiangxi Province Key Laboratory of Laboratory Medicine, Second Affiliated Hospital of Nanchang University, Nanchang, China; ^5^Department of Rheumatology and Immunology, First Affiliated Hospital of Nanchang University, Nanchang, China

**Keywords:** early gastric cancer, lymph node metastasis, machine learning, predictive model, regularized dual averaging (RDA)

## Abstract

**Objective:**

This study aimed to establish the best early gastric cancer lymph node metastasis (LNM) prediction model through machine learning (ML) to better guide clinical diagnosis and treatment decisions.

**Methods:**

We screened gastric cancer patients with T1a and T1b stages from 2010 to 2015 in the Surveillance, Epidemiology and End Results (SEER) database and collected the clinicopathological data of patients with early gastric cancer who were treated with surgery at the Second Affiliated Hospital of Nanchang University from January 2014 to December 2016. At the same time, we applied 7 ML algorithms—the generalized linear model (GLM), RPART, random forest (RF), gradient boosting machine (GBM), support vector machine (SVM), regularized dual averaging (RDA), and the neural network (NNET)—and combined them with patient pathological information to develop the best prediction model for early gastric cancer lymph node metastasis. Among the SEER set, 80% were randomly selected to train the models, while the remaining 20% were used for testing. The data from the Second Affiliated Hospital were considered as the external verification set. Finally, we used the AUROC, F1-score value, sensitivity, and specificity to evaluate the performance of the model.

**Results:**

The tumour size, tumour grade, and depth of tumour invasion were independent risk factors for early gastric cancer LNM. Comprehensive comparison of the prediction model performance of the training set and test set showed that the RDA model had the best prediction performance (F1-score = 0.773; AUROC = 0.742). The AUROC of the external validation set was 0.73.

**Conclusions:**

Tumour size, tumour grade, and depth of tumour invasion were independent risk factors for early gastric cancer LNM. ML predicted LNM risk more accurately, and the RDA model had the best predictive performance and could better guide clinical diagnosis and treatment decisions.

## Introduction

Gastric cancer ranks as the fifth most common malignant tumour and third in mortality worldwide (1, 2). Early gastric cancer (EGC) is defined as lesions confined to the mucosa and submucosa, regardless of size or lymph node metastasis (3).

EGC treatment is being gradually replaced by more minimally invasive methods, such as endoscopic mucosectomy and endoscopic submucosal dissection (4, 5). Compared with gastrectomy, endoscopic treatment has the advantages of a short operation time, less trauma, faster recovery, and fewer complications (6–8). The main risk of minimally invasive endoscopic treatment is lymph node metastasis (LNM), which severely affects the prognosis of patients, and lymph node dissection is required for patients with LNM (9, 10). According to reports, the rate of LNM in EGC is 10–25.3% (9, 11). Endoscopic treatment of EGC patients with LNM undoubtedly increases the risk of recurrence. Therefore, an accurate prediction of the possibility of LNM in EGC before surgery can better guide clinical decision-making.

Presently, studies have reported the risk factors for lymph node metastasis in EGC and have established predictive models. However, these results regarding certain risk factors for lymph node metastasis were inconsistent (12, 13). Because of the complexity of medical data, important connections exist between the various factors of the prediction model, and visible differences are observed in the calculation methods of the model. Machine learning (ML) algorithms are methods that can accurately process raw data, analyse the connections among important data, and make accurate decisions (14, 15). Compared with traditional regression methods, ML algorithms are characterized by their superior performance in predicting results within large databases (16, 17). Currently, considering the complexity and hugeness of medical data, machine learning algorithms have critical application value in assisting disease diagnosis and predicting clinical outcomes (18, 19). Liu et al. established an RF model using machine learning to accurately predict the risk of bone metastasis in thyroid cancer patients (20). Using machine learning and comparing six machine learning algorithms, Zhu et al. finally established an XGBoost model with the best performance in predicting the occurrence of central lymph node metastasis for papillary thyroid cancer patients, helping patients better determine the scope of surgery (21).

Therefore, this study used ML to compare the efficacy of different prediction models for LNM of EGC to identify an accurate prediction method and accurately guide the selection of clinical diagnosis and treatment plans.

## Methods

### Study Population

**Figure 1:**
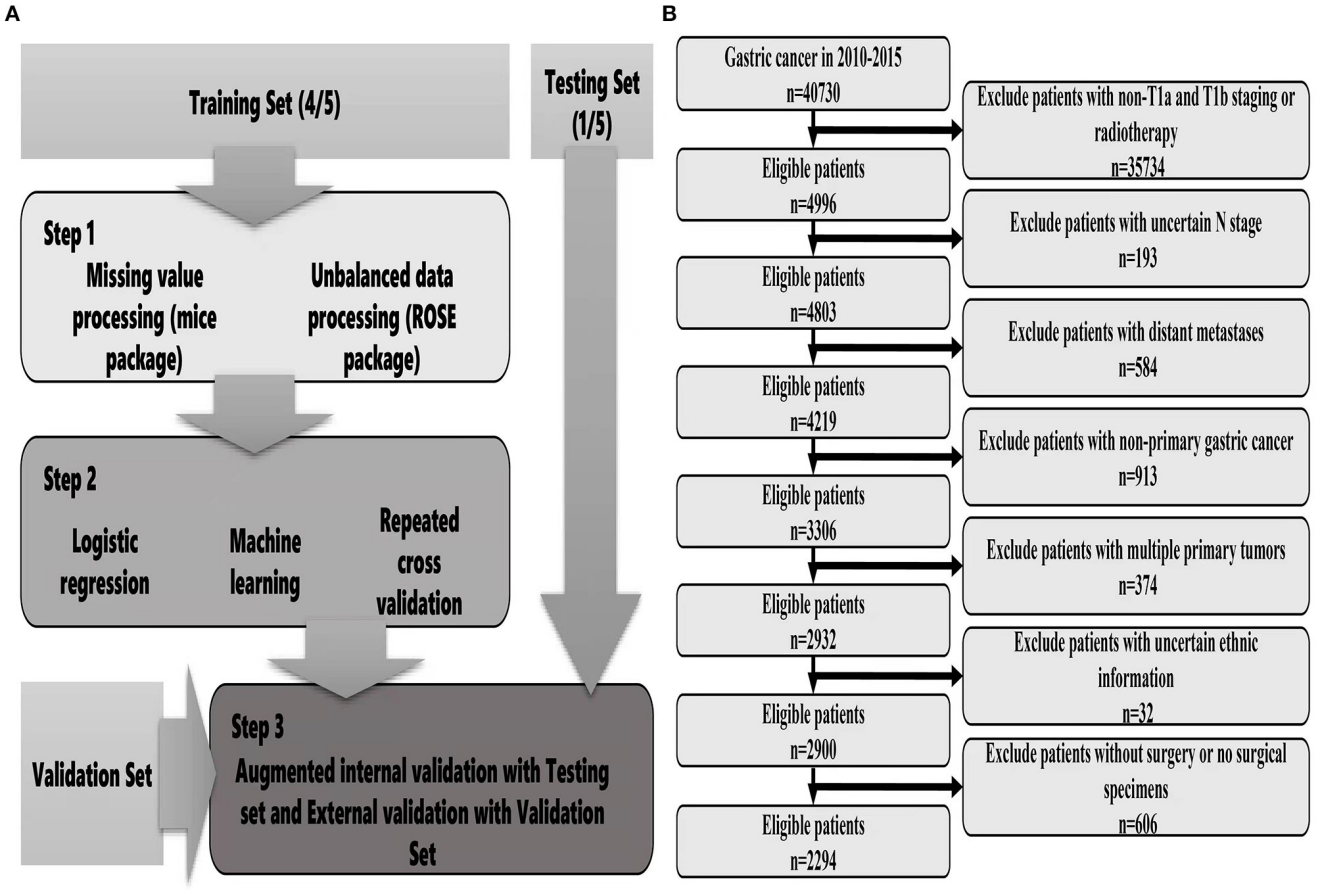
Flow chart of data screening and statistical analysis [**(A)** statistical analysis; **(B)** data screening].

The data were obtained from the Surveillance, Epidemiology and End Results (SEER) database of the National Cancer Institute, which covers basic information for ~28% of US cases. The collection of patient information did not require informed consent because this information was publicly available (account number: 12,846-Nov 2019). From the database, we mainly collected relevant information, including general characteristics, clinical tumour characteristics, pathological characteristics, treatment methods, survival and prognosis. We also collected the clinical data of EGC patients who were treated with surgery from January 2014 to December 2016 at the Second Affiliated Hospital of Nanchang University. The inclusion criteria were as follows: (1) Patients undergoing surgical treatment; (2) A pathological diagnosis of early gastric cancer; (3) Complete survival information. The exclusion criteria were as follows: (1) Multiple tumours *in situ*; (2) Distant metastasis; (3) Incomplete tumour staging; (4) Incomplete information. The tumour site, grade, and histology were coded according to the International Classification of Diseases for Oncology, version 3. Tumour stage was coded according to the AJCC tumour–node–metastasis staging system, 7th edition (22). The detailed screening process is shown in Figure 1B.

### Data Classification

Gastric cancer patients were diagnosed from 2010 to 2015. The ages were grouped as follows: <50 years, 50–60 years, 60–70 years, 70–80 years, and >80 years. Race was grouped as follows: White, Black, and other (American Indian/AK Native, Asian/Pacific Islander). Tumour size was divided as follows: <2 cm, 2–5 cm, >5 cm and NA. Tumour grade was divided as follows: Grade I, Grade II, Grade III, Grade IV, and NA. Tissue classification included signet ring cell carcinoma and non-signet ring cell carcinoma. The depth of tumour invasion included T1a and T1b. The location of the primary tumour was grouped as follows: cardia, fundus, gastric body, antrum, pylorus, lesser curvature, greater curvature, and overlapping/NOS.

### Statistical Methods

For descriptive statistics, chi-squared test or Fisher's exact probability method was used to compare categorical variables. Binary logistic regression was used to analyse the risk factors for lymph node metastasis of EGC. The results were represented by odds ratios (ORs) and 95% confidence intervals (CIs) using the caret package (R software, version 4.1.0) and establishment of different prediction models. Missing values were detected using the mice package and filled with predictive mean matching. The database patients were randomly divided into a training set and a test set at a ratio of 8:2, and hospital patients were used as the external verification set. The training set was used for model development, and the test set was used for evaluation and verification. Considering that the proportion of patients with LNM was too low, we used the ROSE package to balance the training set. Since then, 7 types of ML algorithms have been established by the training set, including the generalized linear model (GLM), RPART, random forest (RF), gradient boosting machine (GBM), support vector machine (SVM), regularized dual averaging (RDA), and the neural network (NNET). During the training process, 10 cross-validations were performed for each model to maintain the stability of the models, and the best hyperparameters were selected using random search. In the test set, the F1-score value, AUROC, sensitivity and specificity of each model were used to comprehensively evaluate the model, compare the performance differences of different prediction models, and conduct difference testing. Finally, the independent external verification set was used to further validate the accuracy and generalization ability of the best prediction model (Figure 1A).

## Results

### General Characteristics

According to our inclusion and exclusion criteria, the SEER database involved 2,294 patients, including 1,839 cases in the training set and 458 cases in the test set. The LNM rate of the training set was 14.5%. The LNM rate of the test set was 14.4%.A total of 227 cases were identified in the external validation set, and the LNM rate was 51.1%. Table 1 displays the clinical and pathological variables of the SEER dataset and external validation set.

**Table 1:** Clinical and pathological characteristics of SEER date and validation set.

**Variable**	**SEER date**		**Validation Set**		***P***
	**LNM (%) NO (1,961)**	**LNM (%) YES (333)**	**LNM (%) NO (111)**	**LNM (%) YES (116)**	**value**
**Age (years)**					
<50 50–60 60–70 70–80 >80	153 (80.1%) 321 (84.4%) 568 (85.9%) 577 (85.1%) 342 (89.0%)	38 (19.9%) 59 (15.6%) 93 (14.1%) 101 (14.9%) 42 (11.0%)	32 (56.1%) 49 (59.8%) 20 (33.9%) 10 (35.7%) 0 (0.0%)	25 (43.9%) 33 (40.2%) 39 (66.1%) 18 (64.3%) 1 (100.0%)	*P* < 0.001 *P* < 0.001 *P* < 0.001 *P* < 0.001 *P* < 0.001
**Race**					
White Black Others	1,257 (87.2%) 224 (80.2%) 480 (83.6%)	184 (12.8%) 55 (19.8%) 94 (16.4%)			
**Tumor size (cm)**					
<2 2–5 >5 NA	908 (89.8%) 603 (78.6%) 78 (62.4%) 372 (95.1%)	103 (10.2%) 164 (21.4%) 47 (37.6%) 19 (4.9%)	44 (84.6%) 65 (42.2%) 2 (9.5%)	8 (15.4%) 89 (57.8%) 19 (90.5%)	*P* = 0.232 *P* < 0.001 *P* < 0.001
**Grade**					
I II III/IV NA	335 (95.4%) 656 (86.8%) 710 (77.5%) 260 (95.5%)	16 (4.6%) 99 (13.2%) 206 (22.5%) 12 (4.5%)	2 (100.0%) 61 (55.0%) 48 (42.1%)	0 (0.0%) 50 (45.0%) 66 (57.9%)	*P* = 0.911 *P* < 0.001 *P* < 0.001
**Organization type**					
SRC NSRC	288 (82.1%) 1,673 (86.1%)	63 (17.9%) 270 (13.9%)			
**Depth**					
T1a T1b	1,083 (94.5%) 878 (76.4%)	62 (5.5%) 271 (23.6%)	57 (68.7%) 54 (37.5%)	26 (31.3%) 90 (62.5%)	*P* < 0.001 *P* < 0.001
**Sex**					
Female Male	1,196 (85.3%) 765 (85.6%)	205 (14.7%) 128 (14.4%)	36 (56.2%) 75 (46.0%)	28 (43.8%) 88 (54.0%)	*P* < 0.001 *P* < 0.001
**Primary site** Cardia Fundus Body Antrum	627 (88.8%) 49 (89.0%) 209 (83.2%) 558 (83.7%)	79 (11.2%) 6 (11.0%) 42 (16.8%) 108 (16.3%)			
Pylorus Lesser curve Greater curve Overlapping/NOS	47 (73.4%) 185 (85.6%) 75 (84.3%) 211 (85.4%)	17 (26.6%) 31 (14.4%) 14 (15.7%) 36 (14.6%)			

### Analysis of Risk Factors for LNM in EGC Patients

First, univariate analysis showed that race, tumour size, tumour grade, tumour tissue type, tumour location, and depth of tumour invasion were related to LNM, and the results were statistically significant (*P* < 0.05) (Table 2). We conducted binary logistic regression analysis on factors *P* < 0.1 (age, race, tumour size, tumour grade, tumour tissue type, tumour site, and depth of invasion). Tumour size, tumour grade and infiltration depth were independent risk factors for LNM in patients with EGC (Table 3). The external validation set also confirmed that tumour size, tumour grade, and depth of invasion were risk factors for lymph node metastasis (*P* < 0.05) (Table 4).

**Table 2:** General characteristics and lymph node metastasis in the SEER database.

**Variable**	**Total (n% 2,294)**	**lymph node metastasis (n%) NO (1,961)**	**lymph node metastasis (n%) YES (333)**	***P* value**
Age (years)				0.063
<50 50–60 60–70 70–80 >80	191 (8.3%) 380 (16.5%) 661 (28.8%) 678 (29.5%) 384 (16.9%)	153 (80.1%) 321 (84.4%) 568 (85.9%) 577 (85.1%) 342 (89.0%)	38 (19.9%) 59 (15.6%) 93 (14.1%) 101 (14.9%) 42 (11.0%)	
Race				0.004
White	1,441 (62.8%)	1,257 (87.2%)	184 (12.8%)	
Black	279 (12.1%)	224 (80.2%)	55 (19.8%)	
Others	574 (25.1%)	480 (83.6%)	94 (16.4%)	
Sex				0.843
Male Female	1,401 (61.0%) 893 (39.0%)	1,196 (85.3%) 765 (85.6%)	205 (14.7%) 128 (14.4%)	
Tumor size (cm)				<0.001
<2 2–5 >5 NA	1,011 (44.0%) 767 (33.4%) 125 (5.4%) 391 (17.2%)	908 (89.8%) 603 (78.6%) 78 (62.4%) 372 (95.1%)	103 (10.2%) 164 (21.4%) 47 (37.6%) 19 (4.9%)	
Grade				<0.001
I II III/IV NA	351 (15.3%) 755 (32.9%) 916 (39.9%) 272 (11.9%)	335 (95.4%) 656 (86.8%) 710 (77.5%) 260 (95.5%)	16 (4.6%) 99 (13.2%) 206 (22.5%) 12 (4.5%)	
Organization type				0.047
SRC NSRC	351 (15.3%) 1,943 (84.7%)	288 (82.1%) 1,673 (86.1%)	63 (17.9%) 270 (13.9%)	
Depth				<0.001
T1a T1b	1,145 (56.3%) 1,149 (43.7%)	1,083 (94.5%) 878 (76.4%)	62 (5.5%) 271 (23.6%)	
Primary site				0.017
Cardia Fundus Body Antrum Pylorus Lesser curve Greater curve Overlapping/NOS	706 (30.8%) 55 (2.4%) 251 (10.9%) 666 (29.1%) 64 (2.7%) 216 (9.4%) 89 (3.9%) 247 (10.8%)	627 (88.8%) 49 (89.0%) 209 (83.2%) 558 (83.7%) 47 (73.4%) 185 (85.6%) 75 (84.3%) 211 (85.4%)	79 (11.2%) 6 (11.0%) 42 (16.8%) 108 (16.3%) 17 (26.6%) 31 (14.4%) 14 (15.7%) 36 (14.6%)	

**Table 3:** Multivariate analysis of the risk of LNM in the SEER database.

**Variable**	**Total (n% 2,294)**	**OR (95%CI)**	***P* value**
Age (years)			0.055
<50 50–60 60–70 70–80 >80	191 (8.3%) 380 (16.5%) 661 (28.8%) 678 (29.5%) 384 (16.9%)	2.199 (1.286–3.760) 1.705 (1.072–2.713) 1.480 (0.972–2.253) 1.412 (0.936–2.129) 1 (Reference)	0.004 0.024 0.068 0.100 −
Race			0.084
White Black Others	1,441 (62.8%) 279 (12.1%) 574 (25.1%)	1 (Reference) 1.518 (1.039–2.218) 1.204 (0.888–1.634)	− 0.031 0.232
Tumor size (cm)			<0.001
<2 2–5 >5 NA	1,011 (44.0%) 767 (33.4%) 125 (5.4%) 391 (17.2%)	1 (Reference) 1.765 (1.330–2.343) 4.313 (2.742–6.785) 0.749 (0.437–1.285)	− <0.001 <0.001 0.294
Grade			<0.001
I II III/IV NA	351 (15.3%) 755 (32.9%) 916 (39.9%) 272 (11.9%)	1 (Reference) 2.273 (1.295–3.992) 3.984 (2.290–6.933) 1.660 (0.743–3.708)	− 0.004 <0.001 0.217
Depth T1a T1b	1,145 (56.3%) 1,149 (43.7%)	1 (Reference) 4.108 (2.994–5.636)	<0.001
Organization type SRC NSRC	351 (15.3%) 1,943 (84.7%)	1 (Reference) 0.945 (0.657–1.358)	0.758
Primary site			0.386
Cardia Fundus Body Antrum Pylorus Lesser curve Greater curve Overlapping/NOS	706 (30.8%) 55 (2.4%) 251 (10.9%) 666 (29.1%) 64 (2.7%) 216 (9.4%) 89 (3.9%) 247 (10.8%)	1 (Reference) 0.780 (0.308–1.976) 1.069 (0.679–1.681) 1.074 (0.748–1.542) 2.160 (1.059–4.404) 0.813 (0.493–1.343) 0.836 (0.424–1.650) 0.963 (0.602–1.540)	− 0.601 0.774 0.699 0.034 0.419 0.606 0.874

**Table 4:** General characteristics and lymph node metastasis of the external verification group.

**Variable**	**Total (n% 227)**	**lymph node metastasis (n%) NO (111)**	**lymph node metastasis (n%) YES (116)**	***P* value**
Age (years) <50 50–60 60–70 70–80 >80	57 (25.1%) 82 (36.1%) 59 (26.0%) 28 (12.3%) 1 (0.5%)	32 (56.1%) 49 (59.8%) 20 (33.9%) 10 (35.7%) 0 (0.0%)	25 (43.9%) 33 (40.2%) 39 (66.1%) 18 (64.3%) 1 (100.0%)	0.005
Tumor size (cm) <2 2–5 >5	52 (22.9%) 154 (67.8%) 21 (9.3%)	44 (84.6%) 65 (42.2%) 2 (9.5%)	8 (15.4%) 89 (57.8%) 19 (90.5%)	<0.001
Grade I II III/IV	2 (0.9%) 111 (48.9%) 114 (50.2%)	2 (100.0%) 61 (55.0%) 48 (42.1%)	0 (0.0%) 50 (45.0%) 66 (57.9%)	0.036
Depth T1a T1b	83 (36.6%) 144 (63.4%)	7 (68.7%) 54 (37.5%)	26 (31.3%) 90 (62.5%)	<0.001
Sex Female Male	64 (28.2%) 163 (71.8%)	36 (56.2%) 75 (46.0%)	28 (43.8%) 88 (54.0%)	0.165

### Model Performance in Predicting LNM

**Figure 2:**
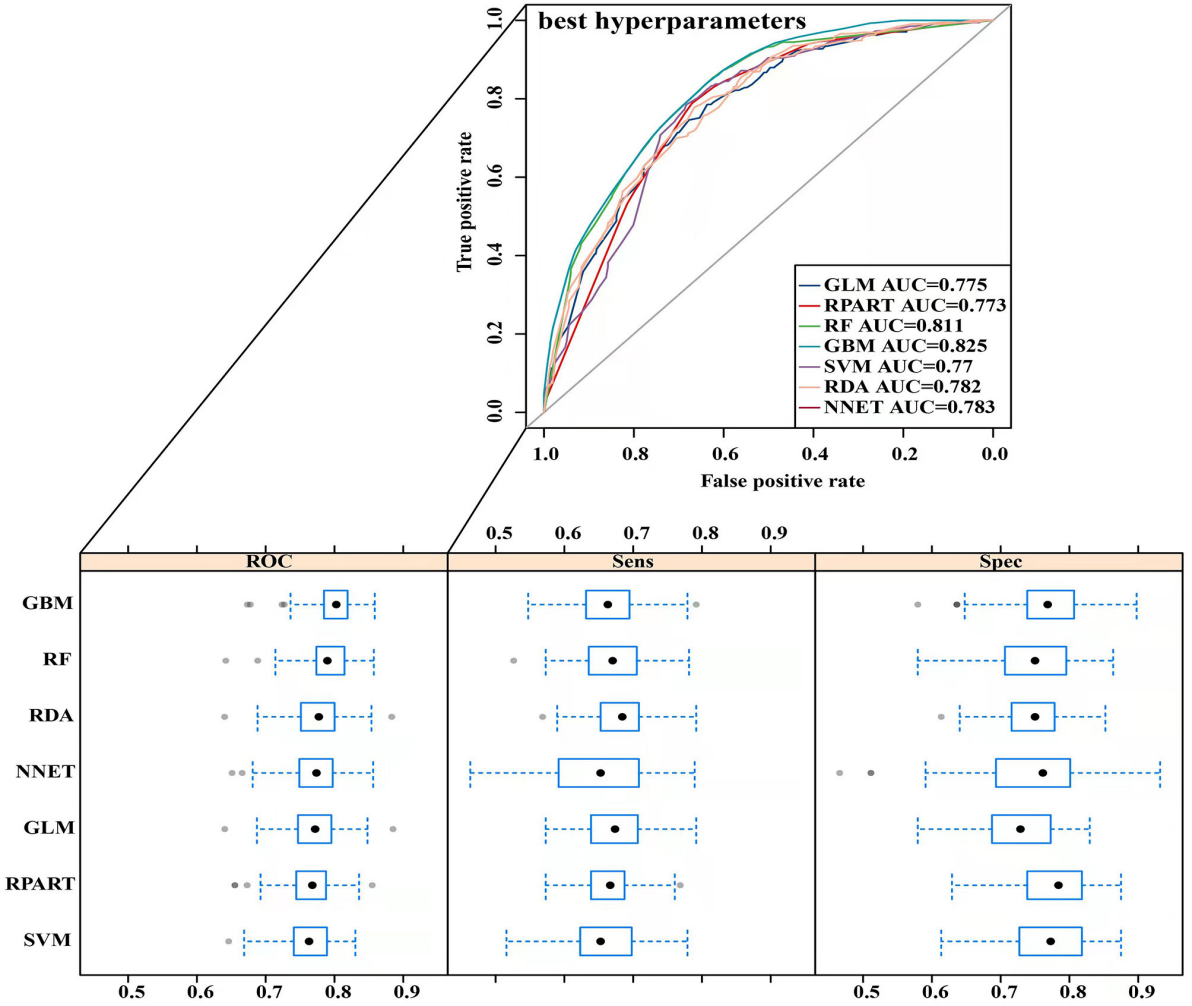
Receiver operating characteristic (ROC) curve of the training set prediction model.

**Figure 3:**
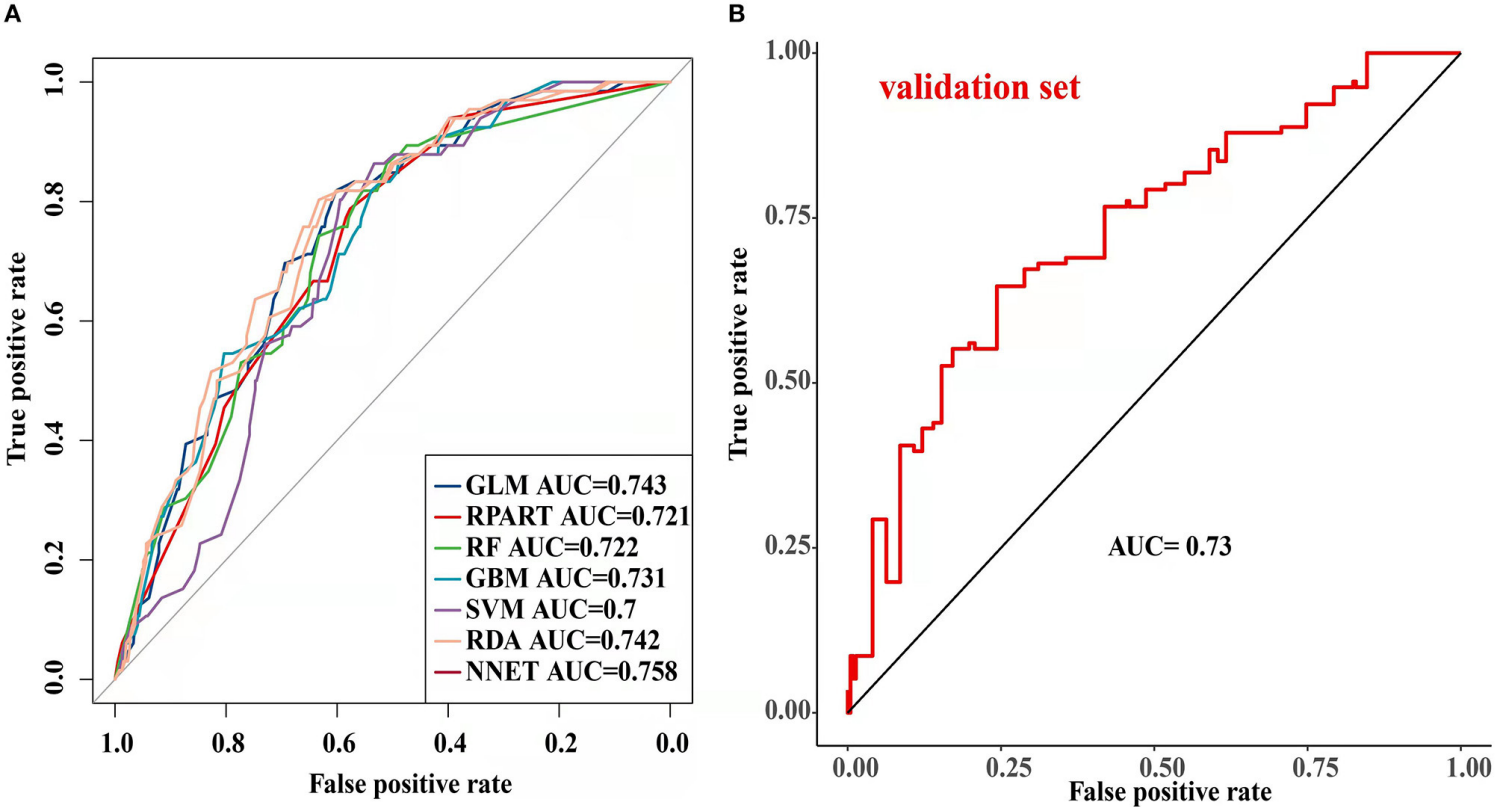
Receiver operating characteristic (ROC) curve of the testing set and validation set prediction model [ **(A)** testing set; **(B)** validation set].

The parameters of the training set were adjusted to balance the model and avoid overfitting the model. After balancing the parameters of the training set, we found that the GBM model had the best predictive ability, with AUCROC = 0.825 (Figure 2). The AUCROC of all the models in the test set was >0.7, where NNET had the highest AUCROC (0.758) and SVM AUCROC (0.7) was the lowest (Figure 3). The F1-score value was suitable to evaluate the predictive performance of unbalanced samples. In the test set, RDA had the best predictive performance, which was significantly better than that of GBM (F1-score: 0.773, sensitivity (recall): 0.661, specificity: 0.712; F1-score: 0.731, sensitivity (recall): 0.607, specificity: 0.682). Based on these results, RDA was selected as the best model to predict LNM (Table 5). At the same time, we collected 227 patients from the Second Affiliated Hospital of Nanchang University from January 2014 to December 2016 as an external validation set to verify the applicability of the RDA prediction model (AUCROC = 0.73). Therefore, we believe that the RDA model is robust in predicting LNM (Figure 3).

**Table 5:** Comparison of prediction performance of different models to LNM.

**Models**	**F1-score**	**Sensitivity (Recall)**	**Specificity**
GBM	0.731	0.607	0.682
GLM	0.771	0.656	0.727
NNET RDA	0.729 0.773	0.592 0.661	0.818 0.712
RF RPART	0.763 0.737	0.648 0.615	0.727 0.682
SVM	0.748	0.633	0.652

## Discussion

Endoscopic mucosal resection and endoscopic submucosal dissection have been widely used to treat EGC and have been established as the standard method to treat early upper gastrointestinal tumours in Japan (23). According to the guidelines of the Japanese Gastric Cancer Association (JGCA), well-differentiated, non-ulcerated intramucosal carcinoma with a diameter <2 cm is the absolute indication for endoscopic therapy, while ulcerated and undifferentiated submucosal carcinomas are the expanded indications (24). For patients at risk of LNM, radical surgery is still recommended. Therefore, accurate prediction of the risk of LNM in patients with EGC before surgery is extremely important for the choice of clinical treatment methods. Presently, the sensitivity and specificity of endoscopic ultrasonography and CT and other imaging examinations to determine EGC lymph node metastasis are not ideal (25, 26).

In recent years, studies have shown that tumour size, tumour grade, depth of invasion, nerve invasion, and ulcers are risk factors for LNM in patients with EGC, and a prediction model has been established (27, 28). However, because of the complexity and large size of the various factors of the data and differences among the calculation methods of the models, the importance of the factors in the prediction model and prediction performance were also significantly different. Mu et al. (29) established an LNM prediction model by logistic regression and showed that lymphatic vascular invasion, differentiation type, tumour diameter and T stage were independent risk factors, with model AUC = 0.861 and validation set AUC = 0.911. Lin et al. (28) found that female sex, tumours larger than 20 mm, submucosal invasion and histological types of undifferentiated tumours were independent risk factors, with model AUC = 0.694 and validation set AUC = 0.796. Both studies used the same logic calculation method, but the model performance was quite different, and the results were different. To resolve this issue, we used the most advanced ML algorithms to compare the performance differences among various prediction models and selected the prediction model with the best performance.

In the present study, we used univariate and binary logistic regression analyses to show that tumour size, depth of invasion, and tumour grade were independent risk factors for LNM in EGC, a finding that was consistent with most research reports (30). We found that when the tumour was a poorly differentiated or undifferentiated submucosal tumour with a size >2 cm, the rate of lymph node metastasis increased 2–4 times. To more accurately predict the risk of LNM and screen the best predictive models, we constructed 7 predictive models using ML algorithms and compared them. First, the training set was modelled, revealing that the GBM model showed the best predictive performance both before and after data balancing (Figure 2). The GBM model had the advantages of high accuracy and fast speed and showed evident preponderance in processing many features; however, it had the disadvantage of overfitting. We evaluated 7 models using the test set and used the F1-score value, sensitivity, and specificity to reflect the effectiveness of the model. The F1-score value is an index to measure the accuracy of a two-class model, considering both the accuracy and recall of the model. Evaluating the test set showed that the RDA model had the best predictive performance, which was significantly better than that of the GBM model. In summary, we believe that the GBM model may show overfitting in the training set making it unsuitable for the data in the test set; however, the RDA model had the best predictive performance. Similarly, the external validation set confirmed that the RDA model was the best predictive model for LNM in EGC and was applicable to the Eastern population (AUC = 0.73).

Our model contained three important factors: tumour size, tumour grade and depth of tumour invasion. In previous reports, tumour diameter affected lymph node metastasis in early gastric cancer, and the larger was the tumour diameter, the higher was the risk of LNM in patients (31). This phenomenon may be due to larger tumour diameters invading the surrounding tissues more easily. Our study also confirmed this important feature. Milhomen et al. (32) found that undifferentiated tumours and submucosal infiltration were closely related to LNM of EGC, a finding that was consistent with our findings. Poorly differentiated and deeply infiltrating tumours may have sufficient nutritional support because cancer cells invade surrounding tissues, capillaries and lymphatic vessels; thus, they have the potential for faster growth and metastasis. We used these three critical factors to construct the best RDA model, which could better predict the risk of LNM in EGC help clinicians make accurate diagnosis and treatment plans and avoid overtreatment.

The present study used seven predictive models based on machine learning and the SEER database to compare the performance of different predictive models to obtain the model with the best predictive performance plus clinical data as external verification. We comprehensively verified the calculation methods used in most studies in recent years to establish the LNM prediction model for EGC and obtained the best prediction model. To our best knowledge, this report is the first to use ML to explore the establishment of the best LNM prediction model for EGC.

However, this study has several limitations. First, because of the scant clinicopathological information in the database, fewer influencing factors were identified in the model. Second, the number of samples finally included in this study was small, leading to certain limitations in machine learning that are more suitable for large sample data. The small sample size is a relatively common problem, and how to solve this problem is the focus of future research, for which we will continue to work. Finally, although we corrected the sample imbalance problem in the SEER dataset as much as possible, this problem may still interfere with the results and affect the generalization ability of the model.

## Conclusions

In summary, we compared the performance of seven prediction models using ML algorithms, among which the RDA model had the best performance. The model included three important predictors—tumour size, tumour grade, and depth of tumour invasion—and the external validation set also showed that the model had accurate predictive capabilities and some applicability. The study findings can better help doctors make clinical diagnoses and allow patients to benefit from better treatment.

## Data Availability Statement

The datasets presented in this study can be found in online repositories. The names of the repository/repositories and accession number(s) can be found in the article/supplementary material.

## Author Contributions

HT and ZZ conceived and designed this study. HY and HL collected and assembled the data. HT and ZN analysed and interpreted the data. HT, ZN, and HL drafted the manuscript. CH and JL prepared the figures and tables. All the authors read and approved the final manuscript.

## Funding

This report was supported by the National Natural Science Foundation of China (Grant Numbers: 81860433 and 82103645), the Natural Science Youth Foundation of Jiangxi Province (Grant Number: 20192BAB215036), Jiangxi Province Natural Science Key R&D Project-General Project (Grant Number: 20202BBG73024) and Training Plan for Academic and Technical Young Leaders of Major Disciplines in Jiangxi Province (Grant Number: 20204BCJ23021).

## Conflict of Interest

The authors declare that the research was conducted in the absence of any commercial or financial relationships that could be construed as a potential conflict of interest.

## Publisher's Note

All claims expressed in this article are solely those of the authors and do not necessarily represent those of their affiliated organizations, or those of the publisher, the editors and the reviewers. Any product that may be evaluated in this article, or claim that may be made by its manufacturer, is not guaranteed or endorsed by the publisher.
